# Supplementary material for: Bioprocessing of Two Crop Residues for Animal Feeding into a High-Yield Lovastatin Feed Supplement
Source: Animals (Basel). 2022 Oct 7;12(19):2697. doi: 10.3390/ani12192697 (PMC9559462; doi:10.3390/ani12192697)
Supplement: Supplementary file 1 [file animals-12-02697-s001.zip › animals-1924907-supplementary.pdf]

Supplementary Material

Bioprocessing of two crop residues for animal feeding into a high-yield lovastatin feed supplement

Contents **Page**

Annex 1. Criteria to improve the research on solid substrate fermentation of agricultural residues/feedstocks for lovastatin production SM2

Annex 2. Analysis of final concentrations of lovastatin as well as zero-order kinetic rate coefficients of increase of lovastatin in the cultures SM6

Annex 3. Efficiency of the solid-sated fermentation, degradation efficiencies of lignin and (cellulose + hemicellulose) fractions, and index ε SM7

Annex 4. Proofs of Equations Eq 2 and 3 in the text of the MS SM9

Annex 5. Complementary Tables SM10

References SM13

Annex 1. Criteria to improve the research on solid substrate fermentation of agricultural residues/feedstocks for lovastatin production

Criteria 1. Strain availability

The availability of the strains used in experimental work is a key element in the reproducibility of biotechnological scientific research. This, in turn, is an important issue in the scientific method [57].

If the strains are not available from a recognized international or institutional agency from which other scientists and industry can obtain/order the strains, an investigation cannot be replicated, thus a basic requirement of the scientific method is violated. This, in turn, can compromise the reproducibility and reliability of the results of a scientific paper [58].

Criteria 2. Proximal analysis of substrates

Proximal analysis of substrates before and after the SSF bioprocess is an important part of the research profile. It is also a criterion to determine whether the processing of agricultural residues has valorized them (levels of the desired metabolite production, nutritional value of the processed residue, etc.). From point of view of animal science, the objective for any feeding program is to achieve an appropriate balance of feed ingredients to satisfy the nutritional needs of the animals [59]. Thus, proximal analysis of the post-fermented substrates accomplishes this goal.

Criteria 3. The degradation efficiency of substrates

It allows discriminating the preferential use of the degradable components (cellulose, hemicellulose, and lignin) of the agricultural residues. On the other hand, it would be interesting

to know if the digestibility of lignocellulosic substrates was improved through the breakdown of the plant cell wall carbohydrates by fungi [59].

It also allows for internal quality control of the experiment results. For example, if there is significant disappearance of lignin when the strain used is not ligninolytic, it is a wake-up call to review the experiment for possible systematic errors, microbiological contamination, etc.

Criteria 4. Statistical analysis of the results

The statistical analysis of results is also a must in modern research [60]. It allows determining whether differences between results obtained in different conditions "treatments" are significant or not, and then making correct and reliable decisions about the choice of variables and levels for future application, research, scaling up, etc. Other functions of statistics in science, for instance, include translating a scientific hypothesis into one or a set of statistical hypotheses and interpreting the experimental results in terms of the hypotheses and their consequences to theory and practical applications [60]. Also, statistical analysis will permit a determination of the degree of uncertainty or bound the uncertainty that characteristically is associated with experimental research.

Criteria 5. Kinetics of Lv production

The kinetic modeling of lovastatin production allows to identify lags of fermentation, the apparent order of reaction in terms of the product (lovastatin), and therefore the probable order of reaction in terms of degradable substrates, as well as the useful time of fermentation. The latter in turn is related to the recommended duration of fermentation (assuming a batch process), which should not be exceeded, and which will save the size and/or run time of bioreactors where the *SSF* is conducted. Also, the performance of different types of bioreactors, and, in the end, bioreactor/process selection, depending on the kinetics, among other features [48].

Criteria 6. Characterization of organic compounds from post-fermented substrates

The production of certain organic compounds by SSF of agricultural residues could cause toxic effects in livestock. For instance, mycotoxins could be a threat to the animal's immune and organ systems (kidney and liver). Also, the animal feed intake is adversely affected by mycotoxin-contaminated animal feeds. In some cases, the residues of mycotoxins in edible foods (e.g., meat or milk) can negatively affect human health [23].

Interestingly, organic compounds present in post-fermented materials could exhibit beneficial effects. As an example, the production of Lv and other statins (simvastatin) from agricultural residues could mitigate the methane emissions from ruminal fermentation [4, 5].

Thus, the characterization of organic compounds from post-fermented substrates, particularly fungal secondary metabolites after *SSF* bioprocess is essential since it can offer additional insight into the possible toxic effects of post-fermented agricultural residues. Yet, only 1 (ours) out of 8 papers in Table 1 of the MS have made the effort to characterize the organic compounds present in post-fermented materials.

In summary, the previous papers published in the open literature relevant to our topic of research do not meet all these criteria. Only 2 papers out of 8 reported that their strains were available from established Culture Collections [12, 14].

Only one article in 8 previous works reported the composition of the substrates and fermented materials [12]. On the other hand, considering the 7 previous works, none of these investigations presented the characterization of the organic compounds present in post-fermented materials. Regarding results on the degradation efficiencies of cell wall components and again considering the 7 previous works, only one article has presented useful information.

Thus, we proposed an *ad hoc* score based on these 6 criteria for each work, with 0 if it is missing, or 1 if the criterion was considered. This gives a possible maximum of 6. A quick inspection of Table 1 shows that there was only one work with a score of 3 [12]; the highest, followed by two works with a score of 2 (Syed et al. [9]; Munir et al. [14]); two with 1 (Bashir et al. [13] and Pansuriya et al. [10]); two with 0 (Patil et al. [8] and Gulyamova et al. [11]).

Interestingly, this translates into an average score of 3.2/6 of the previous relevant literature. This confirms that the scope of previous research is very limited regarding the criteria above discussed, and there is room for improving the research on Lv production by SSF of agricultural residues. Our present article is one attempt to gain insight more comprehensively on several issues that are essential for a better understanding of SSF of agricultural residues that produce lovastatin.

Annex 2. Analysis of final concentrations of lovastatin as well as zero-order kinetic rate coefficients of increase of lovastatin in the cultures

An *ANOVA* of the 2^2 factorial experiment considering only the final concentrations of Lv at 16-day incubation was also carried out [61] followed by the corresponding test of means.

A kinetic model of zero-order in Lv yield (*Lv* final concentration in this case) was fitted to data [48].

$$C = C_o + k*t = b_o + b_1*t \quad \text{(SM2.1)}$$

where C_o stands for the initial concentration of *Lv* (at time 0 day; in mg g^{-1} DM_{fed}), k is the zero-order rate constant (mg (g DM_{fed}*day)$^{-1}$); b_o is the intersection at the origin of the linear regression equation ($b_o = C_o$); b_1 is the slope coefficient of the linear regression ($b_1 = k$).

With more precision, only the points and corresponding triplicates that fell in the linear range of lovastatin increase were used for the fitting. Linear regressions were performed using Excel for Office 365, Data analysis/Regression. Values of the slope coefficients b_1 were subjected to an ANOVA of a simple 2^2 factorial experiment (two strains x two substrates) and a test of means based on Tukey's test [61].

Annex 3. The efficiency of the solid-sated fermentation, degradation efficiencies of lignin and (cellulose + hemicellulose) fractions, and index ε

The efficiency of SSF (E_{SSF}) which is the lignin degradation compared to 'cellulose + hemicellulose' breakdown, was calculated according to Shrivastava et al. [39] as given by Eq. SM3.1:

E_{SSF} (%) = [(loss of lignin) / (loss of cellulose plus hemicellulose)] * 100 (SM3.1)

We defined an *ad hoc* indicator (ε) according to the Eq. SM3.2

$$\varepsilon = \eta_{lig} / \eta_{(c+h)} \quad (SM3.2)$$

where η_{lig} and $\eta_{(c+h)}$ are degradation efficiencies of lignin and 'cellulose + hemicellulose' in oat straw, respectively.

This indicator would shed light on the issue of whether the degradation of 'cellulose + hemicellulose' was higher than that of lignin, or not. Since the strains of *A. terreus* used in our work are not reported to be lignin-degraders, values of $\varepsilon < 1$ are expected.

The degradation efficiencies of lignin and 'cellulose + hemicellulose' in oat straw were estimated by Eqs. SM3.2 and SM3.4, respectively:

$$\eta_{lig} = 1 - (\gamma_{ligf} / \gamma_{ligi}) * [(1 - \gamma_{ligi}) / (1 - \gamma_{ligf})] \quad (SM3.3)$$

$$\eta_{(c+h)} = 1 - (\gamma_{(c+h)f} / \gamma_{(c+h)i}) * [(1 - \gamma_{(c+h)i}) / (1 - \gamma_{(c+h)f})] \quad (SM3.4)$$

where γ_{ligi} and $\gamma_{(c+h)i}$ are the 'lignin' and 'hemicellulose plus hemicellulose' contents in the material, respectively. Correspondingly, γ_{ligf} and $\gamma_{(c+h)f}$ are the final contents of such parameters. All the contents in these equations are in kg kg^{-1} of *DM*.

As a note to the Reader, the Eq SM3.3 and SM3.4 correspond to Eq 2 and 3 in the manuscript.

Annex 4. Proofs of Equations (Eq 2 and 3) in the text of the MS

Let the subindex j represent either the parameter lignin or 'cellulose plus hemicellulose. In the following, Msi is the initial dry matter content of the substrate; Msf is the final dry matter of the substrate; γ_{ji} is the initial content of parameter j (either lignin or (cellulose + hemicellulose) in kg/kg dry matter in the substrate); γ_{jf} is the final content of parameter j (either lignin or (cellulose + hemicellulose), in kg kg^{-1} dry matter in the substrate.)

The first equation for the degradation efficiency of parameter j is given by Eq. SM4.1.

$$\eta_j = (Msi * \gamma_{ji} - Msf * \gamma_{jf})/(Msi\ \gamma_{ji}) = [(1 - (Msf/Msi)*(\gamma_{jf}/\gamma_{ji})] \quad (SM4.1)$$

The development of overall and parameter mass balances leads to

$$Msf = Msi -(Msi * \gamma_{ji} - Msf * \gamma_{jf}) \quad (SM4.2)$$

$$Msf = Msi - Msi\ \gamma_{ji} + Msf\ \gamma_{jf} \quad (SM4.3)$$

$$Msf\ [1- \gamma_{jf}] = Msi\ (1 - \gamma_{ji}) \quad (SM4.4)$$

$$Msf = Msi\ [(1 - \gamma_{ji})/(1 - \gamma_{jf}) \quad (SM4.5)$$

Rearranging the ratio Msi/Msf from Eq SM4.5 and substituting into the 2nd term Eq. SM4.1, and performing some algebra leads to

$$\eta_j = 1- [(Msi/ Msf)\ (1- \gamma_{ji}/ 1- \gamma_{jf})\ (\gamma_{jf} /\gamma_{ji})] = 1 - (\gamma_{jf} / \gamma_{ji}) * [(1- \gamma_{ji}) / (1 - \gamma_{jf})] \quad (SM4.6)$$

$$\eta_j = 1 - (\gamma_{jf} / \gamma_{ji}) * [(1- \gamma_{ji}) / (1 - \gamma_{jf})] \quad (SM4.7)$$

Annex 5. Complementary Tables

Table SM5.1. Analysis of variance of the slopes b_1 of the zero-order kinetic model of lovastatin concentrations

Dependent variable: fitted value of the slope b_1 (mg/(gDM*day))					
Source	DF[a]	Sum of squares	Mean square	F Value[b]	Pr>F[c]
Model	3	1.29547917	0.43182639	68.82	0.007
Error	4	0.02510005	0.006227501		
Total	7	1.32057922			

Source	DF	Tipo III SS	Mean square	F Value	Pr>F
A[d]	1	0.4258722	0.4258722	67.87	0.0012
B[e]	1	0.15523592	0.15523592	24.74	0.0076
A*B[f]	1	0.71437104	0.71437104	113.84	0.0004

[a] degree of freedom; [b] value of the experimental statistic F; [c] p-value; [d] (factor A=Type of strain); [e] (factor B=Type of substrate); [f] interaction of factors A and B

Table SM5.2. Values of the rate coefficients b_1 (slopes) of the zero-order model kinetics fitted in the treatments of this work

Treatment	b_1 [a] mg *Lv* (g *DM**d)$^{-1}$	S.D.[b] mg *Lv* (g *DM**d)$^{-1}$
At[c] H-194, wheat bran	0.5733 b	0.0857
At H-194, oat straw	1.4495 a	0.0869
At H-1976, wheat bran	0.7095 b	0.0849
At H-1976, oat straw	0.3904 b	0.0548

[a] slope coefficient of the regression that fitted zero-order model kinetics to experimental lovastatin concentration; [b] standard deviation of the slope; [c]*Aspergillus terreus.* Means with different letters in a column are statistically different (Tukey; p<0.05).

Table SM5.3. Analysis of variance of final concentrations of lovastatin in our 2^2 factorial experiment. Dependent or response variable: Lovastatin concentration (mg/g DM)

Source	DF[a]	Sum of squares	Mean square	F Value[b]	Pr>F[c]
Model	3	647.5725	215.8575111	47.64	<0.0001
Error	8	36.2449	4.5306167		
Total	11	683.8175			

Source	DF	Tipo III SS	Mean square	F Value	Pr>F
A[d]	1	142.003	142.003	31.34	0.0005
B[e]	1	189.766	189.766	41.89	0.0002
A*B[f]	1	315.803	315.803	68.70	<0.0001

[a] degree of freedom; [b] value of the experimental statistic F; [c] p-value; [d] (factor A=Type of strain); [e] (factor B=Type of substrate); [f] interaction of factors A and B

Table SM5.4. Tukey test for final concentrations of lovastatin in our 2^2 factorial experiment

Treatment	Mean	S.D.[a]
At [b] H-194, wheat bran	6.80 b	2.09
At H-194, oat straw	23.98 a	3.2
At H-1976, wheat bran	9.11 b	1.31
At H-1976, oat straw	5.73 b	1.24

[a] standard deviation; [b]*Aspergillus terreus.* Means with different letters in a column are statistically different ($p<0.05$).

References

All references are available in the main manuscript.
